# Distribution of pathogenicity island (PAI) markers and phylogenetic groups in diarrheagenic and commensal *Escherichia coli* from young children 

**Published:** 2016

**Authors:** Ghazal Naderi, Fakhri Haghi, Habib Zeighami, Fatemeh Hemati, Neda Masoumian

**Affiliations:** 1 *Department of Microbiology, Zanjan University of Medical Sciences, Zanjan, Iran*; 2 *Department of Microbiology, Zanjan Islamic Azad University, Zanjan, Iran *

**Keywords:** Child, Diarrhea, Developing countries, *Escherichia coli*, Pathogenicity Island

## Abstract

**Aim:**

This case–control study investigated the various PAI markers, phylogenetic groups and antimicrobial susceptibility among DEC and commensal *E. coli* isolates.

**Background:**

Diarrheagenic *Escherichia coli* (DEC) is an emerging agent among pathogens that cause diarrheal diseases and represents a major public health problem in developing countries. The major difference in virulence among DEC pathotype and commensals may be related to the presence of specific genomic segments, termed pathogenicity islands (PAIs).

**Patients and methods:**

A total of 600 stool specimens from children (450 with and 150 without diarrhea) were collected and various PAI markers, phylogenetic groups and antimicrobial resistance profile among DEC and commensal *E. coli* isolates were detected.

**Results:**

One hundred sixty eight (90.3%) isolates were resistant to one or more antimicrobial agents. PAI markers were detected in a substantial percentage of commensal (90%) and DEC isolates (99.3%) (*P*> 0.05). The most prevalent PAI marker among DEC and commensal isolates was HPI (91.9% DEC vs. 68% commensal). We found a high number of PAI markers such as SHI-2*, She *and LEE that were significantly associated with DEC. Several different combinations of PAIs were found among DEC isolates. Comparison of PAIs among DEC and commensal isolates showed that many DEC isolates (94.8%) carried two or more PAI markers, while 76% of commensals had only one PAI marker (*P*<0.05). According to the phylogenetic classification, group B2 was the most commonly found in the DEC isolates. Furthermore, our results showed that group B2 can be present in commensal isolates (18%).

**Conclusion:**

These results indicate that PAI markers are widespread among commensal and DEC isolates and these commensal isolates may be reservoirs for transmission of these markers.

## Introduction

 Diarrheal diseases are a leading cause of childhood morbidity and mortality, accounting for approximately 2.5 million deaths annually worldwide (1, 2). Diarrheagenic *Escherichia coli* (DEC) is an emerging agent among pathogens that cause diarrheal diseases and represents a major public health problem in developing countries (3). 

*E. coli* strains can be assigned to one of the main phylogenetic groups: A, B1, B2 or D (4). Strains from groups B2 and D contained more virulence factors than strains from the groups A and B1. The extra-intestinal pathogenic strains usually belong to groups B2 and D, the commensal strains to groups A and B1, whilst the intestinal pathogenic strains belong to groups A, B1 and D (4, 5). Several studies have shown that pathogenic *E. coli* strains may be derived from commensal strains by the acquisition of virulence genes (6). These virulence genes on the chromosome are typically found in specific regions called pathogenicity islands (PAIs) (7). Pathogenicity islands were described for the first time in uropathogenic *Escherichia coli* strain 536 in the late 1980s by Hacker et al (8, 9). PAIs are distinct genetic elements of pathogens encoding various virulence factors such as protein secretion systems, host invasion factors, iron uptake systems, and toxins (10). PAIs are a subset of genomic islands and can be identified by the large size (>10 kb), frequent association with tRNA encoding genes, a G+C content different from host bacterial core genome and etc. These elements are frequently flanked by repeated sequences and carry many fragments of other mobile and accessory genetic elements such as bacteriophages, plasmids and insertion sequence (IS) elements. Some PAIs are unstable regions and can spontaneously disappear from the chromosome. Therefore, PAIs are considered to have evolved from mobile genetic elements by horizontal gene transfer. It can also be assumed that these DNA regions underwent and will continue to undergo further evolutionary changes, resulting in an ongoing evolution of bacterial pathogens (11). Identification of PAIs is essential in understanding the development of disease and the evolution of bacterial pathogenesis (10).

The emergence of antimicrobial resistant *E. coli* has become a serious public health threat worldwide (12). The intensive use of antimicrobial agents in human and veterinary medicine is associated with an emerging resistance against therapeutic drugs, followed by the selection of virulence and resistance gene cassettes carrying *E. coli* strains in humans, animals and the environment (13). Horizontal transfer of these gene cassettes seems to be the main cause of the rapid spread of antibiotic-resistance genes across a wide diversity of bacteria. Beyond the horizontal gene transfer, the loss and acquisition of functional modules are important in the process of rapid bacterial development of resistance (14). So, regular surveillance of antibiotic resistance provides information for antibiotic therapy and resistance control (15).

The objective of the present study was to investigate the distribution of the pathogenicity island (PAI) markers and phylogenetic groups in diarrheagenic and commensal *E. coli* isolated from young children. 

## Patients and Methods

**Patients and bacterial isolation**

In this case-control study, a total of 600 children younger than five years of age voluntarily participated: 450 consecutive patients with and 150 without diarrhea attending three different hospitals in Tabriz, Iran. Patients were enrolled in the study if they had diarrhea and had not taken any antimicrobial agent in the week preceding sampling. Diarrhea was characterized by the occurrence of three or more loose, liquid or watery stools or at least one bloody loose stool in a 24 h period (WHO, 2000). Control subjects were healthy children with no history of diarrhea and antibiotic therapy for at least 1 month. Fresh stool samples collected in Cary–Blair transport medium were cultured on MacConkey agar (Merck, Germany) and one isolate from each patient was identified by conventional biochemical tests. Verified isolates of *E. coli* were preserved at –70 ^º^C in trypticase soy broth (Merck, Germany) containing 20 % (v/v) glycerol for further analysis.

**Table1:** Primers used in this study

PAIs	Marker gene	Oligonucleotide sequence (5ʹ-3ʹ)	Product size bp	Ref.
She	*pic*	fp: ATTCTTCTGGCTGGCATTCC rp: CGGGATTAGAGACTATTGTTGC	606	29
HPI	*irp2*	fp: AAGGATTCGCTGTTACCGGAC rp: TCGTCGGGCAGCGTTTCTTCT	287	29
LEE	*eae*	fp: GACCCGGCACAAGCATAAGC rp: CCACCTGCAGCAACAAGAGG	384	29
SHI-2	*iutA*	fp: GGCTGGACATCATGGGAACTGG rp: CGTCGGGAACGGGTAGAATCG	301	29
Tia	*tia*	fp:CGGGATCCGATGAGAGCAAAACAGGCTT rp: GGGGTACCGAAATGATAAGTTACCCC	756	29
EspC	*espC*	fp: GCTCAACTAAATATTGATAATGTATG rp: CCCAGCCCCAACCCTGAAAC	453	29
O-islands	*efa/lifA*	fp: GAACAAAGAACATTTTCACCAGTTC rp: CTTTCAGGTGGGGAACCCG	521	29
ChuA	*chuA*	fp: GACGAACCAACGGTCAGGAT rp: TGCCGCCAGTACCAAAGACA	279	17
YjaA	*yjaA*	fp: TGAAGTGTCAGGAGACGCTG rp: ATGGAGAATGCGTTCCTCAAC	211	17
TspE4C2	*tspE4C2*	fp: GAGTAATGTCGGGGCATTCA rp: CGCGCCAACAAAGTATTACG	152	17

**Antimicrobial susceptibility testing**

 Susceptibility of isolates to the following antibiotics was examined using the disk diffusion method according to the Clinical and Laboratory Standards Institute (CLSI, 2013) guidelines (16): Amoxicilin (25µg), Aztreonam (30µg), Amikacin (30µg), Cefotaxime (30µg), Cefoxitine (30µg), Ceftazidime (30µg), Ciprofloxacin (5µg), Amoxicillin/ clavulanic acid (30µg), Trimethoprim/ sulfamethoxazole (20+5µg), Cefepime (10µg), Gentamicin (10µg), Imipenem (10µg) and Tetracycline (30µg) (MAST, Merseyside, UK). Isolates shown to be resistant to at least three different classes of antimicrobial agents were determined to be multidrug resistant (MDR). *Escherichia coli* ATCC 25922 was used as a control for antibiotic resistance.

**Detection of PAI markers in** ***E. coli*** **isolates**

All* E. coli* isolates were screened for the presence of O-island, HPI, She, LEE, Tia, SHI-2 and EspC PAIs using the primers listed in Table 1. The following virulence markers were detected on these different PAIs: *irp2* (yersiniobactin encoding gene of HPI); *efa/lifA* (EHEC factor for adherence/lymphocyte inhibitory factor encoding gene of O-island); *pic* (serine protease encoding gene of She); *eae* (intimin encoding gene of LEE); *tia* (toxigenic invasion A encoding gene of Tia), *iutA* (aerobactin receptor encoding gene of SHI-2) and *espC* (enterotoxin encoding gene of EspC). Template DNA was extracted from *E. coli* isolates by boiling. One isolate from each patient was grown in LB (Luria Bertani Broth, Merck, Germany) until the exponential phase with 2 McFarland turbidity. Then, 500 µl of bacterial suspension were centrifuged at 8000 r.p.m for 5 min and pellet suspended in 200 µl sterile deionized water and boiled at 100 ^º^C for 15 min. After centrifugation at 13000 r.p.m. for 3 min, the supernatant was used for PCR. Simplex PCR was performed using DreamTaq PCR Master Mix (Thermo Fisher Scientific), which contains Taq polymerase, dNTPs, gCl_2_ nd the appropriate buffer. Each PCR tube contained 25 µl reaction mixture composed of 12.5 µl of the master mix, 2.5 µl of each forward and reverse primer solution (in a final concentration of 200 nM), 5 µl of DNA with concentration of 400 ng/µl and nuclease-free water to complete the final volume. PCR was performed using the Gene Atlas 322 system (ASTEC). Amplification involved an initial denaturation at 94^°^C, 5 min followed by 35 cycles of denaturation (94^°^C, 1 min), annealing (50^°^C, 45 S for *tia; *54^°^C, 1 min for *espC; *57^°^C, 1 min for *pic; *58^°^C, 1 min for *efa/lifA; *61^°^C, 1 min for *irp-2; *65^°^C, 1 min for *eae; *68^°^C, 1 min for *iutA*) and extension (72^°^C, 1 min), with a final extension step (72^°^C, 8 min). The amplified DNA was separated by submarine gel electrophoresis on 1.5% agarose, stained with ethidium bromide and visualized under UV transillumination.

**Determination of phylogenetic groups**

 Phylogenetic groups were determined using triplex PCR with primers ChuA, YjaA, and DNA fragment TspE4.C2 as described previously (Table 1) (17). *E. coli* isolates were assigned to phylogenetic groups, as follows: *chuA*^+^/*yjaA*^+^, group B2; *chuA*^+^/*yjaA*^−^, group D; *chuA*^−^/ TspE4. C2^+^, group B1 and *chuA*^−^/TspE4.C2^−^, group A.

**Statistical analysis**

 The data were analysed with SSPS version 17.0 software (SPSS). A chi-square test was used to determine the statistical significance of the data. A *P* value of <0.05 was considered significant. 

## Results

**Distribution of PAI markers in commensal and diarrheagenic** ***E. coli*** **isolates**

 A total of 450 children with diarrhea and 150 children without diarrhea were studied. Among the total patients, 133 (22.1%) children were younger than 12 months, 180 (30 %) were 13–24 months and 287 (47.9 %) were 25–60 months. The sex distribution was 364 (60.7%) male and 236 (39.3 %) female. Overall, 186 (31%) *E. coli* isolates were identified in the 600 stool samples: 136 isolates from diarrheal patients and 50 isolates from control group. 

Among the 136 isolates from diarrheal patients, 135 (99.3%) isolates displayed PAI associated sequences, while in the control group, 45 isolates (90%) harbored PAIs (*P*> 0.05).

The frequency of each PAI marker in the patient and control groups is shown in Table 2. Comparison of PAI markers distribution among DEC and commensal isolates showed that DEC isolates harbored markers with a higher frequency than in commensal isolates. The most prevalent PAI marker among DEC isolates was HPI (91.9%), followed by *She *(82.3%), SHI-2 (80.1%) and LEE (79.4%). The frequency of HPI, *She*, SHI-2 and LEE in the healthy controls was 68%, 18%, 14% and 12%, respectively. 

The presence of multiple PAIs with different combinations was found among DEC isolates. Table 3 shows that many of the DEC isolates (94.8%) carry two or more PAIs compared with the commensal isolates (24%) (*P*< 0.05). The number of PAIs per isolate and their specific combinations is shown in Table 3. The mean number of PAIs per isolate was higher in UPEC isolates than commensals (*P*< 0.05). The most frequent combination in DEC isolates was HPI+ *She*+ SHI-2+LEE (50%), followed by HPI+ *She*+ SHI-2 (11%), HPI+ SHI-2+ LEE (7.3%) and HPI+ *She*+ LEE (7.3%). The most commensal isolates (76%) had only one PAI marker. 

**Phylogenetic grouping**

 The distribution of DEC and commensal isolates among the four phylogenetic groups is shown in Table 4. Of the 136 DEC isolates, 58 (42.7 %) fell into group D, 60 (44.1 %) into B2, 13 (9.5 %) into A and 5 (3.7 %) into B1. However, isolates from commensal group revealed a different distribution: A (38%), B1 (30%), B2 (18%) and D (14%). 

**Susceptibility to antimicrobial agents**

 Antimicrobial resistance patterns of isolates are presented in Table 5. In all, 168 (90.3%) isolates were resistant to one or more of the 13 tested antimicrobial agents, with the most frequent resistance found against amoxicillin (73.1%), amoxicillin/clavulanic acid (58.1%), aztreonam (53.8%) and trimethoprim/sulfamethoxazole (49.5%) (Table 5). Imipenem showed the highest activity against isolates and only 2.1% of isolates were imipenem- resistant. A total of 136 (73.1%) isolates were resistant to at least three different classes of antimicrobial agents and considered as multidrug resistant (MDR). The most prevalent MDR pattern was resistance to β-lactams, tetracycline, gentamicin and co-trimoxazole. Prevalence of antibiotic resistance varied among DEC and commensal isolates. Resistance to all antibiotics among patient derived isolates was higher than commensals (Table 5). All MDR isolates of DEC and commensal were positive for the presence of at least one PAI marker. 

**Table 2:** Distribution of PAIs among 50 commensal and 136 diarrheagenic isolates of *E. coli*

Pathogenicity island	No. (%) of isolates carrying PAIs		*P* value	Total (*n*= 186)
	DEC (*n*= 136)	Commensal (*n*= 50)		
HPI	125 (91.9%)	34 (68%)	<0.0001	159 (85.5%)
SHI-2	109 (80.1%)	7 (14%)	<0.0001	116 (62.4%)
*She*	112 (82.3%)	9 (18%)	<0.0001	121 (65%)
LEE	108 (79.4%)	6 (12%)	<0.0001	114 (61.3%)
EspC	11 (8.1%)	4 (8%)	0.984	15 (8%)
Tia	8 (5.9%)	2 (4%)	0.616	10 (5.4%)
O-island	5 (3.7%)	1 (2%)	0.569	6 (3.2%)

**Table 3:** Specific PAI combinations among the 141 *Escherichia coli* isolates (commensal and diarrheagenic) carrying more than one PAI

No. PAIs	PAI Combinations	No. (%) PAIs in DEC (n=136)	No. (%) PAIs in commensal (n=50)	Total No. (%) (n=186)
2 PAIs	SHI-2 + HPI	3 (2.2)	2(4)	17 (9.1)
	She + HPI	2(1.4)	4 (8)	
	LEE + HPI	2 (1.4)	-	
	She + SHI-2	1 (0.7)	-	
	LEE + She	2 (1.4)	-	
	LEE + O-islands	1 (0.7)	-	
3 PAIs	LEE + SHI-2+ HPI	10 (7.3)	-	38 (20.4)
	LEE + She+ HPI	10 (7.3)	-	
	SHI-2 + EspC+ HPI	1 (0.7)	-	
	EspC + O-islands + HPI	-	1 (2)	
	She + SHI-2 + HPI	15 (11)	1 (2)	
4 PAIs	LEE + She + SHI-2+ HPI	68 (50)	2 (4)	76 (40.8)
	She + EspC + Tia + HPI	1 (0.7)	-	
	LEE +She + EspC + HPI	1 (0.7)	-	
	LEE + She + O-islands + HPI	1 (0.7)	-	
	LEE + She + Tia + HPI	1 (0.7)	-	
	She + SHI-2 + O-islands + HPI	1 (0.7)	-	
	LEE + SHI-2 + EspC + HPI	1 (0.7)	-	
5 PAIs	LEE + She + SHI-2 + EspC + HPI	2 (1.4)	1 (2)	8 (4.3)
	LEE + She + SHI-2 + Tia + HPI	2 (1.4)	1 (2)	
	LEE + SHI-2 + EspC + Tia+ HPI	1 (0.7)	-	
	LEE + She + SHI-2 + O-islands + HPI	1 (0.7)	-	
6 PAIs	LEE + She + SHI-2 + Tia + O-islands + HPI	1 (0.7)	-	2 (1)
	LEE + She + SHI-2 + Tia + EspC + HPI	1 (0.7)	-	

**Table 4:** Comparison of phylogenetic groups between DEC and commensal isolates

Group	DEC (n= 136)	Commensal (n= 50)	*P*-value
A	13 (9.5%)	19 (38%)	0.000
B1	5 (3.7%)	15 (30%)	0.000
B2	60 (44.1%)	9 (18%)	0.001
D	58 (42.7%)	7 (14%)	0.000

**Table 5:** Antimicrobial drug resistance patterns among 186 diarrheagenic and commensal *E. coli* isolates

Antimicrobial agents	Commensal *E. coli* (n=50)			Diarrheagenic *E. coli* (n=136)			Total (n=186)		
	R(%) I(%) S(%)			R(%) I(%) S(%)			R(%) I (%) S(%)		
Amoxicillin	31(16.7)	0	19(10.2)	105(56.4)	5(2.7)	26(14)	136(73.1)	5(2.7)	45(24.2)
Cefoxitine	8(4.3)	0	42(22.6)	21(11.3)	0	115(61.8)	29(15.6)	0	157(84.4)
Ceftazidime	12(6.5)	2(1.1)	36(19.3)	57(30.7)	4(2.1)	75(40.3)	69(37.1)	6(3.2)	111(59.7)
Cefotaxime	15(8.1)	3(1.6)	32(17.2)	56(30.1)	4(2.1)	76(40.9)	71(38.2)	7(3.8)	108(58)
Cefepime	5(2.7)	2(1.1)	43(23.1)	48(25.8)	10(5.4)	78(41.9)	53(28.5)	12(6.4)	121(65.1)
Co-amoxiclav	25(13.4)	1(0.5)	24(12.9)	83(44.7)	0	53(28.5)	108(58.1)	1(0.5)	77(41.4)
Imipenem	0	0	50(26.9)	4 (2.1)	0	132(71)	4(2.1)	0	182(97.9)
Azteronam	22(11.8)	5(2.7)	23(12.4)	78(41.9)	11(5.9)	47(25.3)	100(53.8)	16(8.6)	70(37.6)
Gentamicin	12(6.4)	5(2.7)	33(17.7)	50(26.9)	18(9.7)	68(36.6)	62(33.3)	23(12.4)	101(54.3)
Tetracycline	13(7)	1(0.5)	36(19.4)	74(39.8)	1(0.5)	61(32.8)	87(46.8)	2(1.1)	97(52.1)
Co-trimoxazole	20(10.8)	3(1.6)	27(14.5)	72(38.7)	3(1.6)	61(32.8)	92(49.5)	6(3.2)	88(47.3)
Amikacin	5(2.7)	1(0.5)	44(23.7)	35(18.8)	11(5.9)	90(48.4)	40(21.5)	12(6.5)	134(72)
Ciprofloxacin	8(4.3)	3(1.6)	39(21)	43(23.1)	3(1.6)	90(48.4)	51(27.4%)	6(3.2)	129(69.4)

S: susceptible; R: resistant; I: intermediate

% shown for DEC or commensal isolates is % compared to the total number of isolates

## Discussion

Horizontal gene transfer (HGT) seems to be an important mechanism for bacterial evolution and genome complexity and plasticity. PAIs, which are large genomic segments and most likely transferred by HGT, contribute to the virulence and survival of the hosting bacterial strain in a particular environment (18-20). PAIs have been studied widely in the genomes of pathogenic bacteria, but little attention has been given to PAIs in the genomes of commensal members of a species (21). In our study, the presence of various PAI markers and phylogenetic groups was detected among DEC and commensal *E. coli* isolates from the stools of healthy subjects. PAI markers were detected in a substantial percentage of commensal isolates (90%) and DEC isolates (99.3%). According to our results and previous studies, some isolates of *E. coli* from the intestinal tract of healthy people can be considered potentially virulent, as some isolates showed two PAI markers. Already, it has been reported that ExPEC can asymptomatically colonize the intestinal tract (22). Furthermore, there was evidence that the commensal *E. coli* isolates harbored the large number of PAIs (21). However, in our study, commensal isolates contained fewer PAI combinations than DEC isolates. Different studies showed that UPEC isolates harbored PAI markers with significantly higher frequency than commensals (9, 21). In despite to other reports, we detected PAI markers in a substantial percentage of commensal (88 %) and UPEC isolates (98.6 %) in previous our study (23). Distribution of various PAIs (in DEC and commensal isolates) in our study showed the same pattern with other studies (9, 21). High-Pathogenicity Island (HPI), was found most frequently in both commensal and DEC isolates, and is reported to be the most ubiquitous PAI found in Enterobacteriaceae. In previous studies (9, 21), HPI was detected in 38% and 18% of faecal *E. coli* isolates respectively, compared with 68% of isolates in the present study. The high frequency of HPI in commensal isolates has led to the suggestion that HPI may be a fitness island rather than a pathogenicity island (21). Several different combinations of PAI markers were found in 94.8% of DEC isolates, while the most commensal isolates (76%) had only one PAI marker.

Several studies have demonstrated that isolates belonging to phylogenetic group B2 are more commonly extraintestinal pathogenic strains (17, 22). Our results demonstrated that group B2 (44.1%) was the most common group among the *E. coli* isolates from diarrheal patients. The mean number of PAIs accumulated in diarrheal isolates belonging to group B2 was higher than others. In contrast, phylogenetic group A was the most prevalent in the commensal isolates. According to previous reports, isolates belonging to groups A and B1 were more often strictly commensal strains from the intestinal microbiota (17, 21 and 22). However, in our study, 13.2% of DEC isolates belonged to groups A and B1, demonstrating that these groups are also capable of causing diarrheal diseases. The results also showed that group B2, despite being uncommon among commensal isolates, can be present in intestinal flora (18% of our commensal isolates), suggesting that they may act as a reservoir for bacteria that can cause diarrheal diseases.

Antimicrobial resistance among human pathogens has become a major public health problem in developing countries (24). Treatment of infections associated with multidrug resistant *E. coli* is further complicated in Asian countries such as Taiwan, India and Iran (12, 25). In our study, 90.3% of *E. coli* isolates were resistant to one or more antimicrobial agents and 73.1% were multidrug resistant. High frequency of antibiotic resistance among *E. coli* isolates was reported in previous studies in Iran (26-28). The high incidence of amoxicillin (73.1%) and amoxicillin/clavulanic acid (58.1%) resistance in the present study is most probably due to the widespread use of these antimicrobial agents in our country. Furthermore, the loss and gain of resistance genes by mobile genetic elements is an important mechanism in the development of multidrug resistant isolates (14). The high prevalence of antibiotic resistance in DEC isolates may be due to acquisition of the resistance genes from intestinal microbiota as reservoirs for transmission of these genes (29). In conclusion, these results indicate that PAI markers are widespread among commensal and DEC isolates and these commensal isolates may be reservoirs for transmission of these markers.
